# Effect of valproate and pregabalin on human anxiety-like behaviour in a randomised controlled trial

**DOI:** 10.1038/s41398-018-0206-7

**Published:** 2018-08-16

**Authors:** Dominik R. Bach, Christoph W. Korn, Johanna Vunder, Antonia Bantel

**Affiliations:** 1 Clinical Psychiatry Research, Department of Psychiatry, Psychotherapy, and Psychosomatics, Psychiatric Hospital, University of Zurich, 8032 Zurich, Switzerland; 2 Neuroscience Center Zurich, University of Zurich, 8057 Zurich, Switzerland; 3 Wellcome Trust Centre for Neuroimaging and Max Planck-UCL Centre for Computational Psychiatry and Ageing Research, University College London, London, WC1 3BG UK; 4 Institute for Systems Neuroscience, University Medical Center Hamburg-Eppendorf, Martinistrasse 52, 20246 Hamburg, Germany

## Abstract

Valproate is an anticonvulsant drug with strong preclinical evidence for reducing anxiety behaviour in rodents but no clear clinical evidence. To motivate clinical trials, we here investigate the use of valproate in a translational human model of anxiety behaviour. In a double-blind, randomised, placebo-controlled trial, *n* = 118 healthy participants played a previously validated approach/avoidance conflict computer game to measure anxiety-like behaviour, while under 400 mg valproate, under 200 mg of the established anxiolytic/anticonvulsant pregabalin, or under placebo. Saccadic peak velocity and subjective ratings were assessed to control for drug-induced sedation. Compared to placebo, valproate and pregabaline were anxiolytic in the primary outcome, and several secondary outcomes. Bayesian model comparison decisively demonstrated no differences between the two drugs. Subjective and objective sedation was significantly more pronounced under pregabalin than valproate, but did not explain anxiolytic effects. We demonstrate acute anxiolytic properties of valproate in healthy humans. Both drugs have similar anxiolytic properties at the doses used. Valproate is less sedative than pregabalin. Our results suggest clinical trials on the use of valproate in anxiolytic treatment. More generally, we propose a strategy of screening drugs in human preclinical models that can directly be compared across species, such as the approach/avoidance conflict computer game used here. This approach could thus facilitate translational anxiety research.

## Introduction

Rodent anxiety paradigms eliciting approach/avoidance conflict are often used as preclinical models of anxiety disorder^1^. They have successfully enabled screening and development of acutely anxiolytic drugs such as a range of benzodiazepines and other anticonvulsants, all of which act via GABAergic pathways. Of the many non-benzodiazepine drugs tested in rodents, few have made it to the clinic^2^, and this calls for an improved strategy of drug evaluation^3,4^. A discrepancy between pre-clinical and clinical effectiveness could be due to limitations of the rodent models, but also species differences in neurotransmitter systems and signalling pathways. Ideally, research in preclinical human models with cross-species comparability^5,6^, as comparative intermediate step, could motivate, or discourage, the large-scale effort of clinical drug testing.

Pregabalin is an example anticonvulsant that has, after initial rodent experiments^7^, successfully been translated to the clinic in various trials^8–16^. Valproate, on the other hand, is not in standard clinical use for anxiety disorders^17^ despite strong evidence for its anxiolytic properties in rodent tests^18–26^. It acts via a pathway different from benzodiazepines^19,20^, and lacks potential for tolerance^23^, indicating its feasibility for prolonged use. Beyond pilot studies^27^, and trials with bipolar patients and comorbid anxiety^28,29^, only one small randomised control trial (RCT) exists for use in generalised anxiety disorder^30^. The current study sought to answer the question whether valproate has similar acutely anxiolytic properties as pregabalin in humans.

To this end, we capitalised on a preclinical human anxiety model^31^. In this behavioural task, participants forage for monetary tokens in successive epochs under threat of a virtual predator that can take away all collected tokens. This task explicitly pits approach toward rewards (monetary tokens) against avoidance of punishment (capture by predator and loss of previously collected tokens). It thus shares features with several rodent anxiety tests, which are sensitive to benzodiazepines and hippocampus lesions^32^. Our model is not intended to differentiate individuals with or without anxiety disorder, but to temporarily elicit anxiety behaviour in healthy humans. It thus allows focusing on one, possibly transdiagnostic, dimension of psychopathology, in line with calls for abstraction from particular diagnostic frameworks^33^.

Normatively, as the number of collected tokens increases over an epoch of our task, potential loss increases, and participants should become more cautious by retreating to the safe place. We have previously shown that this intra-epoch adaptation of behaviour is reduced under treatment with lorazepam^34^ as well as after hippocampus^31^ and amygdala^34^ lesions. In our previous studies we compared seven measures of intra-epoch adaptation and found that presence in safe place best separated lorazepam from placebo^34^, and also separated patients with hippocampal^31^ and amygdala^34^ lesions from control participants. Hence, presence in safe place was chosen as primary outcome, and the linear drug × time interaction as a priori contrast. We hypothesised that both pregabalin and valproate would reduce the primary outcome in the a priori contrast.

## Materials and methods

### Participants

Participants were recruited from the general population (*n* = 119; 40 per placebo and pregabalin group, 39 in valproate group). One participant in the pregabalin group did not complete the study due to vomiting immediately after drug ingestion, such that *n* = 118 individuals were included in the final analysis. The groups did not differ in age, gender, or baseline personality measures (Table 1). All participants were screened for physical and mental health conditions by a physician (see Table S1 for inclusion and exclusion criteria). The study was conducted in accord with the Declaration of Helsinki and approved by the governmental research ethics committee (Kantonale Ethikkomission Zurich, KEK-ZH 2014-0647) and by the Swiss Agency for Therapeutic Products (Swissmedic, 2016DR2060). All participants gave written informed consent using a form approved by the ethics committee. The study was pre-registered at the primary German Clinical Trials Register (DRKS00010230) and at the Swiss Federal Complementary Database (KOFAM; SNCTP000001772), and conducted at the University of Zurich between 09 June 2016 and 16 September 2016.

**Table 1:** Sample characteristics

Sex	Placebo group	Valproate group	Pregabalin group	*p*
	20 male 20 female	20 male 19 female	20 male 19 female	
	Mean ± SD	Mean ± SD	Mean ± SD	
Age	24.83 ± 4.22	23.97 ± 3.21	23.56 ± 3.17	0.28
STAI X1	34.00 ± 7.55	32.20 ± 5.50	33.27 ± 7.10	0.50
STAI X2	38.41 ± 6.74	37.56 ± 6.40	36.95 ± 6.99	0.63
BDI	3.78 ± 4.25	4.07 ± 3.85	3.67 ± 4.13	0.90
VAS overall	1.72 ± 1.95	1.76 ± 2.12	5.30 ± 2.71	<0.001*
VAS sedation	2.81 ± 2.75	3.12 ± 2.45	6.07 ± 2.83	<0.001*
VAS stimulation	2.77 ± 2.89	2.33 ± 2.46	2.46 ± 2.22	0.74
VAS dizziness	0.92 ± 1.72	1.13 ± 1.72	4.74 ± 3.00	<0.001*
PSV pre	392.6 ± 79.8	391.0 ± 67.1	373.1 ± 79.1	0.46
PSV post	406.5 ± 66.7	393.9 ± 55.8	362.2 ± 69.9	0.009*
Threat rating	51.3 ± 9.8	55.7 ± 9.6	54.9 ± 10.1	0.10
Threat preference	1.48 ± 0.78	1.54 ± 0.68	1.62 ± 0.67	0.68

*STAI X1* trait anxiety score, *STAI X2* state anxiety score, *BDI* depression score, *VAS* visual analogue scale, administered immediately before the computer game, *PSV* peak saccadic velocity from a saccade task before (pre) or after (post) the computer game, *Threat rating* explicit rating of the wake-up probabilities immediately after the computer game, averaged over the three predators (see Table S5 for an analysis of the individual threat levels), *Threat preference* most preferred threat level from three pair-wise comparisons (1: low, 2: medium, 3: high), *p* *p*-value for the omnibus main effect from a 3-level (Placebo, Valproate, Pregabalin) one-way ANOVA

See Table S6 for a covariate analysis accounting for the group differences in VAS and PSV

### Power analysis

To determine required sample size, we conducted a power analysis (using G*power^35^) based on our previous study on lorazepam. This study revealed, in the planned primary outcome and contrast for the present study, an effect size estimate of partial *η*^2^ = 0.0345, based on *t* = 5.34 and df_residual_ = 798. The non-sphericity correction for residual degrees of freedom in this error stratum was Greenhouse–Geissers *ε* = 0.1174, and the average correlation between data points from the same subject in this error stratum was *r* = 0.5081. Based on these sample values, to achieve 80% power at a type I error threshold of *α* = 0.05, a sample size of *n* = 37 per group was needed. We set our target sample size at *n* = 40 per group to allow for attrition.

### Study medication

Our study medication was 400 mg valproic acid (corresponding to 500 mg sodium valproate), supplied in powder form from Katwijk chemie bv (Katwijk, The Netherlands), and 200 mg pregabalin, brand name Lyrica^®^ (Pfizer, Zurich). Study dose for valproate was based on the recommended starting single dose (corresponding to half a daily dose) to minimise side effects. Pregabalin dose was based on previous work in healthy humans^36,37^ and within the range of the manufacturer’s daily dose recommendation on the Swiss market for treatment of GAD (150–600 mg per day in 2–3 single doses). According to manufacturers’ brochures, peak plasma concentrations are reached at approximately 60–90 min (valproate) and 60 min (pregabalin) after oral administration; the drugs’ half-lifes are approximately 10 h (valproate) and 6.5 h (pregabalin). A GMP-licensed pharmacy (Kantonsapotheke Zürich) manufactured four capsules per participant containing 4 × 100 mg valproate, 1 × 200 pregabalin, and 3×mannitol or 4×mannitol (placebo). The pharmacy randomised and blinded the study medication, separately for male and female participants. Randomisation code was broken after the last participant completed the study, and after all data were checked for consistency.

### Procedure

#### Screening visit 1 (day −7 to day −1)

**Fig. 1:**
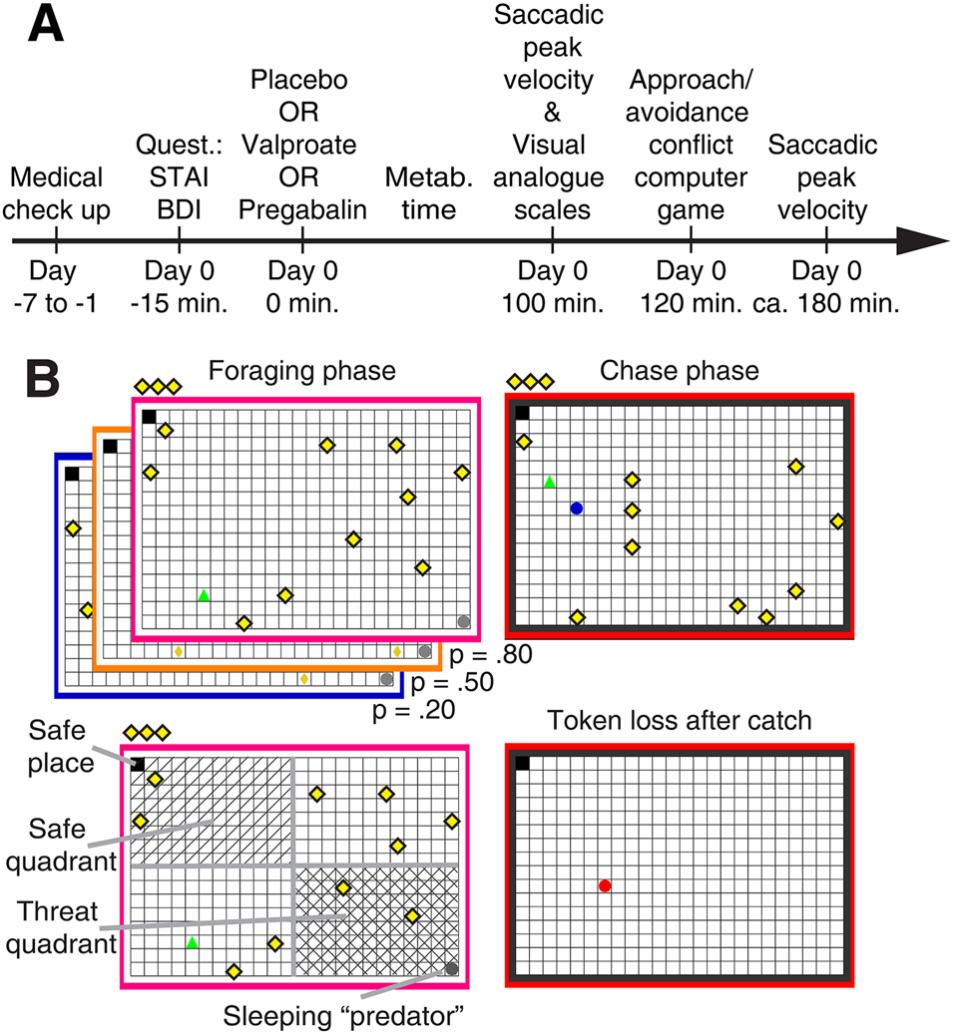
Experimental design. **a** Study procedure. **b** Behavioural approach-avoidance conflict (AAC) computer game. A human player (green triangle) is foraging for tokens (yellow rhombi), which contribute to financial reimbursement at the end of the game. At any time, 10 tokens are present and are replaced in random position when collected. Collected tokens are shown in the upper left corner, above the grid. Meanwhile, a predator (grey circle) is inactive in a corner of the grid, and can attack the human player at any given time according to three probabilities specified by the frame colour. To avoid being caught by the predator, the player can seek shelter in a safe place. Presence in the safe place constitutes the primary outcome measure. The safe place is always diagonal to the initial predator position. During the chase phase, when the predator wakes up, the frame colour turns red. All tokens from this epoch are lost when the player is caught

Exclusion criteria were checked via medical/psychiatric examination, and blood/urine test (Fig. 1a, Supplemental Methods).

#### Drug visit 2 (day 0)

Participants filled in the State-Trait Anxiety Inventory^38^ and Beck’s Depression Inventory^39^ before ingesting study medication. During a 100-min metabolisation time, they were kept under surveillance of study staff. Participants spent this time ad libitum, and were allowed to eat snacks but no meals. Next, we measured saccadic peak velocity^40–42^ (Supplemental Data), and participants filled in visual analogue scales (VAS) to record subjectively perceived overall drug effect (I feel substance effect), sedation (I feel dazed/sleepy), stimulation (I feel activated/awake), and dizziness (I feel dizzy). One hundred twenty minutes after drug ingestions, they started playing the approach/avoidance computer game, which lasted around 1 h. Afterward, we again measured saccadic peak velocity.

### Behavioural AAC paradigm

Participants completed 240 epochs of our previously described AAC task^31,34^, presented using the MATLAB toolbox Cogent (www.vislab.ucl.ac.uk). In each epoch, participants collected monetary tokens on a 24 × 16 grid under threat of being attacked by a predator, which resulted in the loss of all tokens collected in the given epoch (Fig. 1b). One corner of the grid was a safe place, in which the predator could not attack. Location of the safe place was randomised on each epoch.

#### Tokens

At all times, 10 tokens were uniformly distributed on the grid, and every 2 s one of the tokens changed its position randomly. Collected tokens were replaced on the grid, and their number displayed above.

#### Predator

The predator was initially inactive in the corner diagonal to the safe place. The predator could become active and chase participants any time. Colour of the frame around the grid (blue, purple, orange) indicated three distinct predator wake-up probabilities (0.2, 0.5, and 0.8), which participants learned to distinguish. Participants started either in the same place as the predator (which we term “active” condition) or in the safe place, opposite the predator (which we term “passive” condition here). Notably, all rounds entailed going out onto the grid to collect tokens, and over the course of an epoch, behaviour becomes comparable for the two conditions (see Fig. S1).

#### Movements on the grid

Participants coordinated their movements by pressing the four computer keyboard arrow keys. No diagonal movements were possible. Participants could move at a maximum speed of 10 grid blocks per second. All three predators had the same speed of 40 grid blocks per second. Thus, participants could try to escape an active predator by retreating to the safe place but the predator moved four times faster, and thus escape was only possible if participants were close to the safe place.

#### Epoch duration

Epochs lasted 3–15 s (drawn from a uniform distribution with steps of 1.7 s). There was a 3 s countdown with a preview of the grid layout before each epoch, during which the player could not move. This was meant to facilitate orientation with respect to starting place/predator position. After the pre-determined epoch duration, the predator either woke up, or the next epoch started. 240 epochs were divided into five blocks with short self-paced breaks.

#### Post-task questions

Participants rated on a VAS (ranging from 0% to 100%) the wake-up probability and wake-up latency of the three different predators.

#### Bonus epoch

Finally, participants were given the choice to select the predator that they would like to face in a final bonus round. The selection process entailed three consecutive pair-wise comparisons between the predators.

#### Payment

At the end of the game, the average number of tokens from nine randomly selected epochs, plus the bonus epoch, was transformed into a monetary reimbursement that was added to the constant show-up fee.

### Outcome measures

Proportion of presence in safe place (the only grid block which the predator could not enter) was chosen as primary outcome, and the linear drug × time interaction as a priori contrast. As in previous studies^31,34^ we additionally report six further measures as secondary outcomes, Bonferroni-corrected for six comparisons: (1) distance (as the crow flies) from threat (i.e., from the predator), (2) distance from nearest wall, (3) presence in safe quadrant (i.e., the quarter of the grid surrounding the safe place), (4) presence in threat quadrant (i.e., quarter of the grid surrounding the predator position), (5) token collection, and (6) speed when outside safe place. Note that in our previous study on lorazepam, presence in safe quadrant was chosen as primary outcome^34^. This choice had been based on a preceding hippocampus lesion study^31^, but it turned out that presence in safe place better separated lorazepam from placebo^34^. As auxiliary measures, we analyse latency to forage after epoch start, and latency to flight after predator wake-up; catch rates, and the average number of tokens retained at the end of each epoch, including the chase phase; subjectively rated wake-up probabilities and wake-up times of the three different predators, and participants’ preferences in pair-wise comparison of the three predators.

### Statistical analysis

We averaged participants’ positions on the grid within 1 s bins, and then averaged across epochs, for each participant, each condition, and time bin. Since epoch duration was variable, more data were available for earlier than for later time bins. Our factorial design included a between-subjects factor (Placebo/Valproate/Pregabalin) and three within-subjects factors: threat level (wake-up probability, low/medium/high), task (active/passive start), and time (15 time bins of 1 s duration). For some measures (token collection and speed when on grid), some participants had no data values in the final time bins (as they were in the safe place for all trials during these time bins), and these time bins were removed for analysis. We used the software package R (function aov) to perform full multistratum repeated-measures ANOVA model with Greenhouse–Geisser corrected degrees of freedom. We report the following a priori contrasts: (valproate vs. placebo) × time, (pregabalin vs. placebo) × time. To test for drug differences, we also assess the third, non-independent contrast (pregabalin vs. valproate) × time. To confirm drug equivalence, we report a Bayesian model comparison between a model with one predictor per drug, and reduced (non-nested) model with one predictor for placebo, and one for the two drugs. We compute Bayesian information criterion (using the R function BIC) and interpret an absolute BIC difference >6 as decisive^43^.

### Code availability

All codes used to generate the results in this manuscript are fully available from the authors.

## Results

**Fig. 2:**
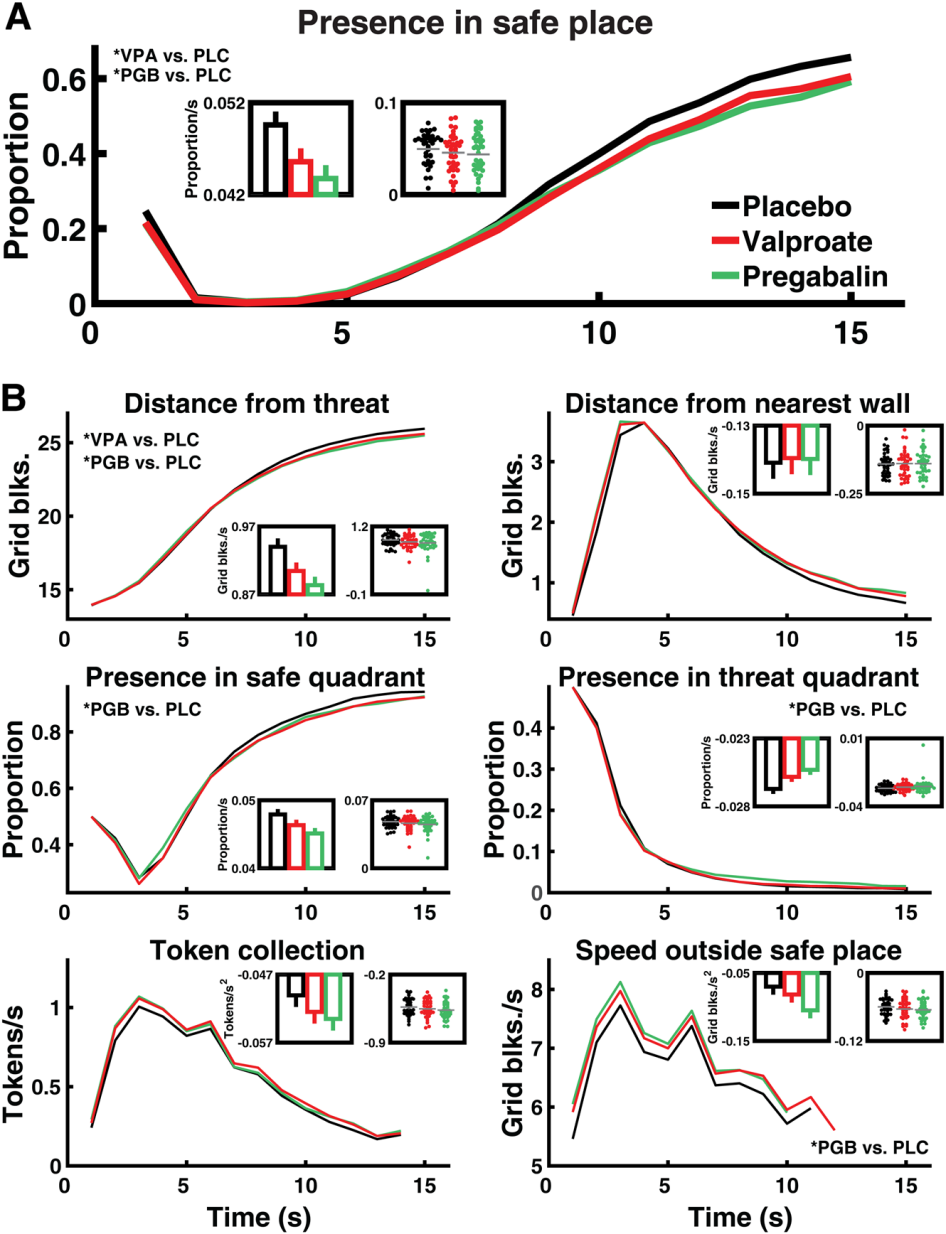
Effect of valproate and pregabalin on primary and secondary outcomes. Line graphs show evolution of outcome measures over 15 s epochs, evaluated in 1 s time bins. Inset bar graphs reflect the a priori contrast and show estimated change over time (linear fixed-effects coefficients ± standard error) for the three conditions. Inset scatter plots show change over time for individual participants, estimated in single-participant models fitted post hoc; these have no relation to the statistical hypothesis test and are shown for illustrative purposes. **a** Proportion of time spent in the safe place (see Fig. 1b) linearly increases over time, and this increase is reduced in participants under valproate or pregabalin as compared to placebo (linear drug × time interaction, see Table 2). **b** Six secondary outcome measures. There is no significant difference between the two drugs in any measure, and Bayesian model comparison favours a model in which they have the same impact as compared to placebo. See Fig. S1 for a comparison with lorazepam. VPA valproate, PGB pregabalin, PLC placebo. *: significant linear drug × time interaction (for secondary outcomes after Bonferroni correction)

Two hours after ingesting valproate, pregabalin, or placebo, participants performed the AAC computer game (Fig. 1b). Both valproate and pregabalin significantly reduced the primary outcome (a priori contrasts: valproate: *F* (1, 185) = 7.56, *p* = 0.007, partial *η*^2^ = 0.005; pregabalin: *F* (1, 185) = 16.08, *p* = 0.001, partial *η*^2^ = 0.010). Figure 2a shows that as intra-epoch time passed, participants under placebo spent increasingly more time in the safe place, and this linear change over time was reduced in participants under both drugs.

In Bonferroni-corrected secondary outcomes, valproate reduced loss adaptation in “distance from threat”, while pregabalin reduced loss adaptation in “distance from threat”, “presence in threat quadrant”, and “speed when on grid” (Fig. 2b). Exploratory analyses of the other error strata are summarised in Table 2. At the same time, participants’ behaviour in the placebo group was generally similar to the behaviour of healthy participants in our previous reports (see Table S2 and Fig. S1).

**Table 2:** Comparison of placebo, valproate, and pregabalin

	Presence in safe place*	Distance from threat	Distance from walls	Presence in safe quadrant	Presence in threat quadrant	Tokens per second	Speed when on grid
	F/t *p*	F/t *p*	F/t *p*	F/t *p*	F/t *p*	F/t *p*	F/t *p*
Valproate	−0.98	−0.58	1.10	−1.05	−0.19	1.27	1.28
	.33	.56	.27	.29	.85	.21	.20
Pregabalin	−1.11	−0.75	1.33	−0.61	0.62	1.13	1.60
	.27	.46	.19	.54	.54	.26	.11
Valproate × threat omnibus	3.43*	3.02	1.60	2.96	1.71	1.98	0.65
	.041	.061	.21	.064	.19	.15	.51
Valproate × threat linear	2.49*	2.04	-1.57	1.95	−1.18	−1.51	−0.87
	.01	.04	.12	.053	.24	.13	.39
Pregabalin × threat omnibus	1.28	1.27	1.43	1.88	0.49	1.76	2.62
	.28	.28	.24	.16	.59	.18	.08
Pregabalin × threat linear	1.56	1.54	−1.67	1.88	−0.35	−1.78	−2.25
	.12	.13	.10	.061	.73	.08	.03
Valproate × task	−1.53	−0.98	−1.05	−1.38	0.98	-0.68	−0.88
	.13	.33	.30	.17	.33	.50	.38
Pregabalin × task	−1.26	−1.31	0.04	−1.04	1.39	1.37	−1.29
	.22	.19	.97	.30	.17	.17	.20
Valproate × time omnibus	0.86	0.75	1.09	0.70	1.43	0.38	0.59
	.40	.50	.36	.55	.23	.75	.55
Valproate × time linear^†^	−2.75*	−2.87*	0.29	−1.97	2.40	−1.42	−0.97
	.007	.005	.77	.050	.017	.16	.33
Pregabalin × time omnibus	1.83	1.90	1.42	2.11	2.35	0.88	1.23
	.17	.14	.23	.097	.074	.44	.29
Pregabalin × time linear^†^	−4.01*	−4.56*	0.24	−3.49*	3.80*	−2.03	−2.93*
	.001	<.001	.81	<.001	<.001	.044	.004
Pregabalin vs. valproate × time linear^‡^	−1.25 .21	−1.68 .094	−0.05 .96	−1.52 .13	1.39 .17	−0.60 .55	−1.95 0.052
Valproate × threat × time omnibus	1.93	1.21	1.31	.91	0.64	0.87	1.12
	.072	.30	.23	.52	.78	.60	.35
Valproate × threat × time linear	5.67*	3.85*	−3.62*	0.60	−2.80*	−2.03	−1.55
	<.001	<.001	<.001	.55	.005	.04	.12
Pregabalin × threat × time omnibus	0.68	0.57	1.09	0.54	0.44	0.46	1.12
	.67	.78	.37	.86	.93	.96	.35
Pregabalin × threat × time linear	3.04*	1.98	−3.17*	1.86	−0.14	−1.27	−3.29*
	.002	.048	.002	.064	.89	.20	.001

Results are shown from a 3 (Group: Placebo, Valproate, Pregabalin) × 3 (Threat: Low, Medium, High) × 2 (Task: Active/Passive) × 15 (Time bins of 1 s each) ANOVA. The table lists *F*-values for omnibus effects and signed *t*-values for polynomial contrasts and for the main effects of drug and task. *p*-values were computed using Greenhouse–Geisser corrected degrees of freedom. Significant *p*-values after Bonferroni correction (primary outcome: *α* = .05; secondary outcomes: *α* = .05/6 ≅ 0.008) are marked with asterisk. Signs of *t*-values are coded as higher-dependent values for drug vs. placebo, higher levels of threat, later time points, and passive vs. active. * primary outcome. † a priori contrasts. ‡ non-independent direct comparison of pregabalin and valproate in the a priori contrast (see also Fig. 2 and Fig. S1)

Next, we directly compared the effect of pregabalin and valproate in a further non-independent contrast (Table 2). No significant difference between the two drugs in their impact on linear time adaptation emerged. To demonstrate drug equivalence, we then conducted a Bayesian model comparison. For the a priori contrast in all outcomes, model evidence decisively favoured a model with a common predictor for both drugs (Table S3), i.e., there was decisive evidence (BIC difference >6) for drug equivalence.

In two performance measures, catch rates and average number of tokens retained at the end of each epoch (including chase phase), no significant group difference emerged (*p* > .1, Table S4). Thus, all participants maximised their token collection, but by doing so they used slightly different strategies.

There was no group difference in explicit ratings of predator probability, or preference for the three predators (Table S4, all *p*’s > .1). After including these measures as covariates (together with their time interaction) into the initial model, the significance of linear drug × time effects remained unchanged (Table S5).

Next, we were concerned that drug-induced sedation might explain the impact of the drugs on the primary and secondary outcome measures. Latency to escape when the predator woke up was different between the study groups (*p* = .029; Table S4), possibly indicating anxiolysis or sedation. Indeed, self-ratings of overall drug effect and sedation were higher for pregabalin than placebo (Table 1, overall: *t* (68) = 6.7, *p* < .001; sedation: *t* (77) = 5.2, *p* < .001) or valproate (overall: *t* (71) = 6.4, *p* < .001; sedation: *t* (74) = 4.9, *p* < .001). Furthermore, saccadic peak velocity, a sensitive measure of drowsiness induced by GABAergic drugs, was lower for pregabalin than placebo (*t* (75) = 2.9, *p* = .005) or valproate (*t* (71) = 2.3, *p* = .033) when measured immediately after the game (Table 1). This is why we included all self-ratings (overall drug effect, sedation, stimulation, dizziness) and saccadic peak velocity before and after the game, into our model as covariates, together with their time interaction. Significance of the a priori contrasts was unchanged in all primary and all secondary outcome measures (Table S5). In sum, although pregabalin appeared to induce relevant sedation in self-ratings and objective measures, we found no evidence that the impact of valproate and pregabalin on loss adaptation in our task could be explained by sedation.

Further exploratory analysis revealed that females responded more strongly to pregabalin than males in primary and one secondary outcome measures while there was no such difference for valproate (Table S6). We note our study was not powered to detect sex-specific drug effects.

Finally, we sought to directly compare the drug doses used here with the effect of 1 mg lorazepam in a previous study^34^ (Table S7 and Fig. S1). All three drugs combined showed an anxiolytic effect on primary outcome as well as on “distance from threat”, “presence in safe quadrant”, “presence in threat quadrant”, and “speed when on grid”. Lorazepam turned out to have a significantly stronger effect than the other two drugs on primary outcome as well as “distance from walls”, “tokens per second”, and “speed when on grid”. In most of these measures, all three drugs were (near-) significantly different from placebo. However, “distance from walls” (potentially measuring thigmotaxis, relating to agoraphobia^44^) was pronouncedly influenced by lorazepam and not by pregabaline or valproate. Thus, it appears not only that the lorazepam dose used was more anxiolytic than the pregabalin or valproate dose, but also that the drugs may somewhat differ in their specific pattern of anxiolytic influences.

## Discussion

Valproate is acutely anxiolytic in various rodent tests^18–26^ but evidence for its efficacy in anxiety disorders is limited to a pilot study^30^. To facilitate further clinical research, we here performed an intermediate comparative research step^5^ and asked whether valproate is acutely anxiolytic in healthy humans. As a main finding, valproate had an anxiolytic effect on the primary and one secondary outcome measure. There was no significant difference between valproate and pregabalin in our a priori contrast in any outcome measure. Bayesian model comparison decisively showed equivalence of the two drugs. Thus, we found no indication of an anxiolytic difference between the two drugs, but sedative side effects were far more pronounced under pregabalin than valproate. Notably, pregabalin dose was chosen based on previous studies in healthy humans^36,37^ and is in the range of the recommended daily doses for treatment of GAD. To minimise side effects, valproate dose was selected more cautiously and is in the range of a half daily starting dose for epilepsy. It is possible that higher doses of valproate have a stronger anxiolytic action but would also elicit more sedative side effects. At the doses used here (200 mg pregabalin, 400 mg valproate), a direct comparison with a previous study^34^ revealed that these drugs were less anxiolytic than 1 mg lorazepam. Furthermore, different from lorazepam, they did not influence thigmotaxis, a behaviour sometimes associated with agoraphobia^44^.

A previous RCT compared 3 × 500 mg valproate (Depakine ® chrono) over 6 weeks with placebo for the treatment of GAD in *n* = 74 patients^30^. This trial found significantly higher response rates (50% HAMD reduction) in valproate vs. placebo-treated patients. As a limitation, placebo responses in this study were much lower than in other studies, e.g., in many RCTs with pregabalin. Until today, no larger phase II studies have been conducted on valproate to replicate this pilot trial. While our results suggest acutely anxiolytic properties of valproate, the single-dose design used here is not directly comparable to prolonged treatment over several weeks or months. Consequently, we cannot directly make statements on clinical effectiveness, which will require new clinical trials.

To test anxiolytic properties, we used our recently established human approach/avoidance conflict paradigm^31,34,45,46^. This paradigm is designed to reflect rodent conflict test and is sensitive to anxiolytic action of lorazepam^34^. It is thus different from tests designed to elicit anxiety feelings, such as public speaking anticipation, for which no unambiguous demonstration of sensitivity to benzodiazepines exists^47^. As a limitation, it is not clear to what extent the precise pattern of benzodiazepine effects in conflict tests reflects their clinical efficacy. Thus, while our approach is likely to reveal drugs that have effects similar to benzodiazepines in our conflict test, they are not guaranteed to have the same effect as benzodiazepines in a clinical condition. This concern could possibly be mitigated by investigating valproate in other preclinical anxiety tests, particularly in tests that are conceptually different from rodent approach/avoidance conflict. While it has been noted that our task uses financial incentives rather than “real” threat^48^ (such as mild electric shocks or unpleasant images and sounds^49,50^), its immersive nature may be effective enough to create a situation comparable with real threat. Specifically, it is the only presently available human approach-avoidance paradigm validated with anxiolytic drugs and lesion models.

Our paradigm was designed to induce behavioural strategies that resemble those seen in clinical anxiety states, such as passive avoidance and behavioural inhibition. Because the diagnosis of anxiety disorders primarily relies on patients’ introspective assessment, i.e., symptoms rather than signs^5^, it would be useful to assess the feelings induced in healthy persons while being engaged in the task presented here, which was not the focus of the current study. At the same time, it may be informative to assess the behavioural strategies that GAD patients use in our task.

Interestingly, the anxiolytic effects of lorazepam, pregabalin, and valproate are probably mediated via the GABAergic system, but by different mechanisms of action. Lorazepam, like other benzodiazepines, binds allosterically to the GABA (A) receptor and thus increases the impact of GABA^51^. The other two drugs increase GABA levels, but by less well understood mechanisms^52^. Pregabalin does not bind to GABA receptors but to the α_2_δ-1 subunit of voltage-gated calcium channels, and this binding is required for the anxiolytic properties of the drug^53^. Valproate inhibits succinic semialdehyde dehydrogenase, such reducing the degradation of succinic semialdehyde, which inhibits the GABA-degrading enzyme GABA transaminase^54,55^. It also acts on non-GABAergic transmission by inhibiting voltage-gated Na channels, and several further mechanisms of action have been speculated, including at the genomic level, but without clear evidence for their relevance even in the main indications for valproate, namely, epilepsy and bipolar disorder^55^. Other antiepileptic drugs potentially acting via GABA-mediated mechanisms are gabapentin, vigabatrin, and tiagabin^52^, which are therefore candidate drugs for future preclinical tests. Once the relevant pathways mediating the anxiolytic action of pregabalin and valproate are elucidated, this could lead to the development of new anxiolytic compounds. We propose all of these substances could be evaluated in our human pre-clinical model, as this would render an investment into large-scale clinical studies more feasible. One concern sometimes raised with respect to rodent approach/avoidance tests is their sensitivity to GABAergic but not other anxiolytic substances such as antidepressants^4^. Investigating the sensitivity of our human model to non-GABAergic anxiolytic as well as non-anxiolytic substances could facilitate the screening of new drugs.

Despite their well-known side effects and addiction potential, benzodiazepines are still commonly prescribed for GAD (e.g., estimated prescription rates in 2002 across the US: 38%^56^) despite the availability of alternatives such as SSRIs, pregabalin, and psychotherapy. While there may be many reasons for this, it could indicate suboptimal response rates to these alternative treatments. A meta-analysis across different drug treatments found response rates between 40 and 75%, with a mean of 55%^57^. Similarly, a recent industry-sponsored combined analysis of pregabalin trials found a response rate (at trial endpoint) of around 60%^58^. This leaves significant room for improvement. It would be interesting whether patients that do not respond to psychotherapy, pregabalin, or SSRIs, could benefit from valproate.

To summarise, our study demonstrates acutely anxiolytic properties of valproate in healthy humans, and thus suggests a potential of this drug in the treatment of anxiety disorders. More generally, we furnish a new strategy for testing drugs in translational anxiety research, by harnessing an intermediate comparative model in healthy humans. This model is not supposed to distinguish individuals with our without anxiety disorders by their behaviour in the test, but instead to temporarily elicit a particular dimension of psychopathology in healthy humans. Thus, a validation of this approach will be the demonstration that effectiveness in the preclinical test predicts clinical efficacy. With this work, we hope to advance clinical research on new treatments for anxiety disorders.

## Electronic supplementary material

Supplemental material

Consort Flow Chart
