# Supplementary material for: Effect of valproate and pregabalin on human anxiety-like behaviour in a randomised controlled trial
Source: Transl Psychiatry. 2018 Aug 16;8:157. doi: 10.1038/s41398-018-0206-7 (PMC6095858; doi:10.1038/s41398-018-0206-7)
Supplement: Supplementary file 1 — Supplemental material [file 41398_2018_206_MOESM1_ESM.pdf]

Supplementary table of contents

Table S1: Participant selection.

Supplementary Methods

Figure S1: Behaviour in placebo group and comparison of Valproate/Pregabalin with 1 mg lorazepam[1].

Table S2: Behaviour of the placebo group.

Table S3: Bayesian comparison of a model using one predictor per drug, and a model using a common predictor for both drugs.

Table S4: Comparison of Placebo, Valproate and Pregabalin in auxiliary measures.

Table S5: Results from ANCOVA to control for possible confounds.

Table S6: Influence of sex on drug effects.

Table S7: Comparison of Valproate/Pregabalin with 1 mg lorazepam[1].

Table S1: Participant selection.

**Inclusion criteria**
Healthy, German-speaking, between 18 and 40 years of age
**Exclusion criteria**
Contraindication to valproate, pregabalin or or mannitol (history of allergic reactions)<br>Drug use in the two weeks prior to the study (exception: contraceptive drugs and incidental use of NSAIDs/paracetamol)<br>Known or suspected use of illicit drugs, use of benzodiazepines, alcohol abuse<br>Any history of psychiatric, neurological, or systemic/rheumatic disease<br>Other clinically significant concomitant disease states<br>Pregnant or breast-feeding women, intention to become pregnant, lack of safe contraception method<br>Participation in any other drug study within the 30 days preceding and during the present study<br>Participation in any previous study with the same approach/avoidance conflict task
**Blood parameters screened**
Blood cell count, electrolytes, C-reactive protein, aspartate aminotransferase (ASAT/GOT), alanine aminotransferase (ALAT/GPT), gamma-glutamyl transferase (gamma-GT), kreatinin, thyroid-stimulating hormone (TSH), free thyroxine (FT4)
**Urine parameters screened**
Amphetamines, barbiturates, benzodiazepines, tetrahydrocannabinol, cocaine, methadone, opioids; women: beta human chorionic gonadotropin (beta-HCG) pregnancy test

Supplementary Methods: Saccadic peak velocity measurement

To control for possible sedative effects, we measured saccadic eye movements as described previously[2]. Measurements were obtained before and after the behavioral AAC paradigm. Eye movements of both eyes were recorded using the EyeLink 1000 System (SR Research, Ottawa, Ontario, Canada) at a sampling rate of 500 Hz. Testing was performed in a dark, soundproof chamber. (In contrast, behavioral testing was performed on a different PC in a room with ambient light.) Participants were seated 57 cm from a monitor (Dell P2012H, 60 Hz refresh rate) with their head position fixed onto a chin rest. Calibration was performed using the 9-point calibration procedure implemented in the EyeLink software. The MATLAB toolbox Psychtoolbox (http://psychtoolbox.org/) was used for task presentation. Participants were asked to follow a target with their eyes (i.e., a black dot with a diameter of 0.3° visual angle on a white background). A trial started with the black dot in the center of the screen (0° visual angle). After a random duration of either 1000 or 2000 ms, the dot jumped to one of four possible peripheral positions (±7.25°, ±14.5°, i.e., left or right of the initial dot position) and remained there for 1000 ms. In total there were 60 trials, i.e., 15 for each position.

The task was analyzed using custom scripts in MATLAB following previously described routines[2]. Only saccades with a minimum amplitude of 1° and a minimum onset latency of 100 ms were included. If a saccade or an eye blink occurred between 100 ms before and after dot movement, trials were excluded. We saccadic peak velocity (maximum value of the movement derivative in degree per second) as the most reliable metric for drug-induced drowsiness[3]. See **Table 1** in main text for results.

References

1 Korn, C. W. *et al.* Amygdala Lesions Reduce Anxiety-like Behavior in a Human Benzodiazepine-Sensitive Approach-Avoidance Conflict Test. *Biological psychiatry* **82**, 522-531, doi:10.1016/j.biopsych.2017.01.018 (2017).

2 Schmechtig, A. *et al.* The effects of ketamine and risperidone on eye movement control in healthy volunteers. *Transl Psychiatry* **3**, e334, doi:10.1038/tp.2013.109 (2013).

3 de Visser, S. J. *et al.* Biomarkers for the effects of benzodiazepines in healthy volunteers. *British journal of clinical pharmacology* **55**, 39-50 (2003).

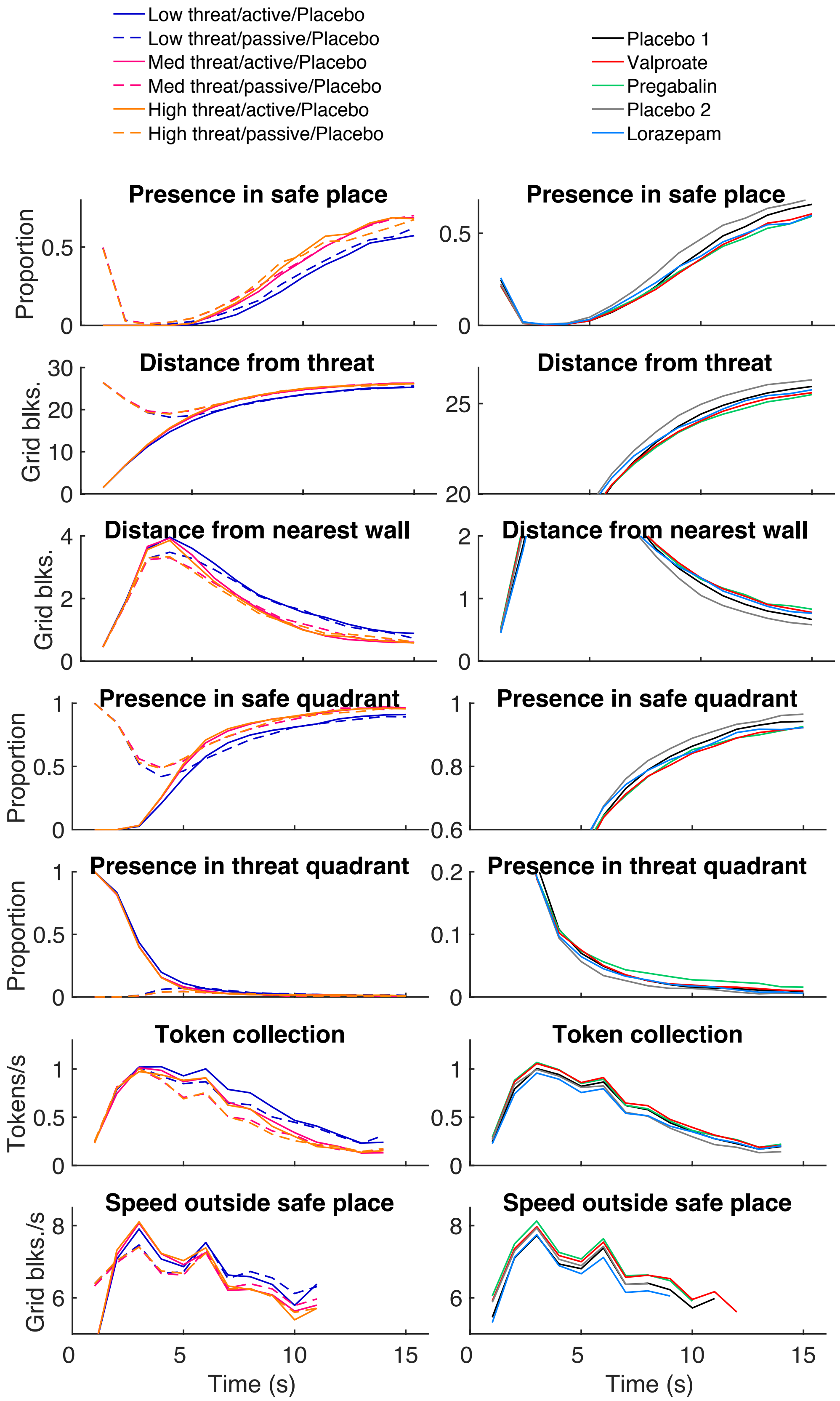

Figure S1: Behaviour in placebo group (left) and comparison of Valproate/Pregabalin with 1 mg Lorazepam (right).

Placebo 1: Placebo group in valproate/pregabalin study. Placebo 2: Placebo group in lorazepam study.

Table S2: Behaviour of the placebo group.

	**Presence in safe place**	**Distance from threat**	**Distance from walls**	**Presence in safe quadrant**	**Presence in threat quadrant**	**Tokens per second**	**Speed when on grid**
	F/t	F/t	F/t	F/t	F/t	F/t	F/t
	P	*p*	*p*	*p*	*p*	*p*	*p*
Threat omnibus	19.24*	17.76*	21.98*	20.57*	4.72	27.78*	15.66*
	< .001	*< .001*	*< .001*	*< .001*	*.01*	*< .001*	*< .001*
Threat linear	5.88*	5.51*	-6.29*	5.86*	-2.71	-6.95*	-5.19*
	< .001	*< .001*	*< .001*	*< .001*	*.008*	*< .001*	*< .001*
Threat quadratic	-1.97	-2.28	2.10	-2.60	1.45	2.69	2.08
	.05	*.03*	*.04*	*.01*	*.15*	*.009*	*.04*
Task	4.09*	9.54*	-3.46*	10.33*	-10.42*	-7.78*	-0.37
	< .001	*< .001*	*.001*	*< .001*	*< .001*	*< .001*	*.71*
Time omnibus	216.42*	592.17*	363.62*	480.76*	794.67*	322.53*	70.58*
	< .001	*< .001*	*< .001*	*< .001*	*< .001*	*< .001*	*< .001*
Threat × task omnibus	0.57	0.32	0.84	0.31	0.19	1.80	4.60
	.57	*.73*	*.43*	*.73*	*.81*	*.17*	*.01*
Task × time omnibus	72.71*	688.74*	16.89*	599.31*	673.28*	14.09*	45.11*
	< .001	*< .001*	*< .001*	*< .001*	*< .001*	*< .001*	*< .001*
Threat × time omnibus	7.86*	6.87*	7.50*	6.58*	2.26	5.37*	5.89*
	< .001	*< .001*	*< .001*	*< .001*	*.02*	*< .001*	*< .001*
Threat × time linear	10.92*	4.75*	-5.05*	4.05*	3.21*	-5.88*	-9.43*
	< .001	*.004*	*< .001*	*.001*	*.001*	*< .001*	*< .001*
Threat × time quadratic	-3.88*	-1.00	3.12*	-2.25	-1.37	2.81*	2.39
	.001	*.32*	*.002*	*.025*	*.17*	*.005*	*.02*
Threat × task × time omnibus	1.05	.96	0.52	0.76	1.45	0.91	1.25
	.40	*.48*	*.94*	*.69*	*.14*	*.59*	*.26*

Results are shown from a 3 (Threat: Low, Medium, High) × 2 (Task: Active/Passive) × 15 (Time bins of 1 s each) ANOVA for n = 40 placebo participants. The table lists F-values for omnibus effects and signed t-values for polynomial contrasts and for the main effect of task. P-values were computed using Greenhouse-Geisser corrected degrees of freedom. Significant p-values after Bonferroni correction (primary outcome: α = .05; secondary outcome: α = .05/6 ≅ .008) are marked with *. Signs of t-values are coded as higher dependent values for higher levels of threat, later time points, and passive vs. active. Shaded column: primary outcome. See also **Figure S1**.

Table S3: Bayesian comparison of a model using one predictor per drug, and a model using a common predictor for both drugs.

Error stratum	Presence in safe place	Distance from threat	Distance from walls	Presence in safe quadrant	Presence in threat quadrant	Tokens per second	Speed when on grid
Drug	4.74	4.74	4.71	4.56	4.11	4.74	4.66
Drug × task	4.69	4.66	3.58	4.65	4.60	0.60	4.60
Drug × threat	9.84	9.73	10.57	9.95	9.98	10.34	8.92
Drug × time	100.19	98.95	101.59	93.40	97.76	89.04	58.26
Drug × threat × time	204.10	192.67	187.47	208.92	202.98	183.47	120.89

Results are shown for each error stratum of a 3/2 (Group: Placebo, Valproate, Pregabalin/Placebo, Drug) × 3 (Threat: Low, Medium, High) × 2 (Task: Active/Passive) × 15 (Time bins of 1 s each) ANOVA as differences in Bayesian Information Criterion (BIC), referenced to the reduced model (i.e. larger values are better). Absolute BIC differences > 3 can be regarded as decisive, in analogy to a classical p-value of $p < .05$. With one exception (drug × task stratum for tokens per second), evidence is decisively higher for models with collapsed drug predictor, i.e. no difference between the drugs. Shaded column: primary outcome. Shaded rows: error stratum containing a priori contrasts. A null-hypothesis significance test of drug differences is contained in table 2 of the main manuscript.

Table S4: Comparison of Placebo, Valproate and Pregabalin in auxiliary measures.

	Flight latency	Foraging latency	Catch rate	Tokens retained	Subjective wake-up probability	Subjective wake-up time	Choice for last round
Valproate	1.72	-0.76	1.13	1.42	-1.11	0.91	-0.05
	.09	*.45*	*.26*	*.16*	*.27*	*.36*	*.96*
Pregabalin	2.21*	-1.54	0.61	1.95	-0.87	0.77	-0.24
	.03	*.13*	*.54*	*.05*	*.39*	*.44*	*.81*
Valproate × threat omnibus	0.99	0.61	0.23	0.97	1.14	2.15	0.17
	.37	*.54*	*.80*	*.38*	*.32*	*.12*	*.84*
Valproate × threat linear	1.33	-0.82	0.47	-0.93	-0.44	2.07*	0.55
	.18	*.41*	*.64*	*.35*	*.66*	*.04*	*.58*
Pregabalin × threat omnibus	0.23	0.39	0.64	3.20	0.07	0.42	0.12
	.80	*.68*	*.53*	*.04*	*.93*	*.65*	*.88*
Pregabalin × threat linear	-0.58	-0.88	-0.11	-2.53*	0.06	0.10	0.00
	.56	*.38*	*.91*	*.01*	*.95*	*.92*	*> .99*
Valproate × task	-0.89	-0.47	-1.07	1.17			
	.37	*.65*	*.29*	*.24*			
Pregabalin × task	-1.13	0.30	-0.63	0.23			
	.26	*.77*	*.53*	*.82*			
Valproate × threat × task omnibus	0.31	0.91	0.54	0.03			
	.74	*.41*	*.58*	*.97*			
Valproate × threat × task linear	0.78	-0.89	0.76	0.10			
	.43	*.37*	*.45*	*.92*			
Pregabalin × threat × Task omnibus	0.65	0.02	1.66	1.09			
	.52	*.98*	*.19*	*.34*			
Pregabalin × threat × Task linear	-1.00	-0.20	-0.24	-0.51			
	.32	*.84*	*.81*	*.61*			

Results are shown from a 3 (Group: Placebo, Valproate, Pregabalin) × 3 (Threat: Low, Medium, High) [× 2 (Task: Active/Passive)] ANOVA. The table lists F-values for omnibus effects and signed t-values for polynomial contrasts and for the main effects of group and task. Significant p-values without Bonferroni correction are marked with *. Signs of t-values are coded as higher dependent values for drug vs. placebo, higher levels of threat, and passive vs. active.

Table S5: Results from ANCOVA to control for possible confounds.

Contrast	Covariate	Presence in safe place	Distance from threat	Distance from walls	Presence in safe quadrant	Presence in threat quadrant	Tokens per second	Speed when on grid
		t	t	t	t	t	t	t
Valproate x time linear	*Age*	-3.13*	-3.47*	0.92	-2.43	2.89*	-1.03	-1.09
	STAI X1	-2.92*	-2.70*	0.42	-2.01	1.94	-1.11	-0.46
	STAI X2	-2.79*	-2.76*	0.33	-1.94	2.17	-1.28	-0.66
	BDI	-2.58*	-2.84*	0.18	-1.97	2.59	-1.56	-1.15
	VAS overall	-2.79*	-3.00*	0.31	-2.02	2.51	-1.41	-1.00
	VAS sedation	-3.20*	-3.52*	0.44	-2.35	2.88*	-1.37	-1.21
	VAS stimulation	-2.74*	-2.73*	0.26	-1.90	2.16	-1.36	-0.89
	VAS nausea	-2.94*	-3.24*	0.33	-2.14	2.75*	-1.52	-1.10
	PSV pre	-2.74*	-2.85*	0.29	-1.95	2.38	-1.43	-0.95
	PSV post	-2.70*	-2.92*	0.34	-2.04	2.47	-1.53	-0.98
	Threat rating	-3.70*	-3.08*	0.42	-1.78	2.42	-2.04	-1.79
	Threat preference	-2.90*	-2.96*	0.34	-2.01	2.33	-1.36	-0.95
Pregabalin x time linear	*Age*	-4.54*	-5.42*	1.16	-4.16*	4.50*	-1.44	-3.09*
	STAI X1	-4.08*	-4.48*	0.29	-3.50*	3.64*	-1.90	-2.74*
	STAI X2	-4.07*	-4.37*	0.31	-3.44*	3.40*	-1.77	-2.41
	BDI	-4.13*	-4.56*	0.28	-3.51*	3.79*	-1.99	-2.91*
	VAS overall	-5.54*	-8.59*	1.56	-5.94*	7.44*	-1.29	-4.24*
	VAS sedation	-7.34*	-9.32*	1.62	-6.46*	7.42*	-1.35	-4.76*
	VAS stimulation	-3.99*	-4.46*	0.22	-3.44*	3.63*	-1.98	-2.87*
	VAS nausea	-5.94*	-8.55*	0.78	-5.37*	7.68*	-3.19*	-4.37*
	PSV pre	-4.26*	-4.73*	0.46	-3.47*	3.74*	-2.25	-2.76*
	PSV post	-3.97*	-4.81*	0.56	-3.66*	3.98*	-2.42	-2.89*
	Threat rating	-4.80*	-4.72*	0.34	-3.32*	3.81*	-2.54	-3.60*
	Threat preference	-4.32*	-4.75*	0.34	-3.59*	3.64*	-1.88	-2.89*

The table lists t-values for the a priori contrasts (linear drug x time interaction) from 3 (Group: Placebo, Valproate, Pregabalin) × 3 (Threat: Low, Medium, High) × 2 (Task: Active/Passive) × 15 (Time bins of 1 s each) ANCOVA with different covariates. P-values were computed using Greenhouse-Geisser corrected degrees of freedom to account for violations of multisphericity. Significant p-values after Bonferroni correction (primary outcome: α = .05; secondary outcome: α = .05/6 ≅ .008) are marked with *. Signs of t-values are coded as higher dependent values for drug vs. placebo, higher levels of threat, later time points, and passive vs. active. Shaded column: primary outcome. See shaded rows in **Table S1** for the corresponding ANOVA without covariates.

Table S6: Influence of sex on drug effects.

	**Presence in safe place** F/t *P*	**Distance from threat** F/t *p*	**Distance from walls** F/t *p*	**Presence in safe quadrant** F/t *p*	**Presence in threat quadrant** F/t *p*	**Tokens per second** F/t *p*	**Speed when on grid** F/t *p*
Sex × Valproate	0.25<br>.80	1.07<br>.29	-0.85<br>.40	0.75<br>.45	-1.15<br>.25	-1.15<br>.25	-0.21<br>.83
Sex × Pregabalin	-1.12<br>.27	-1.44<br>.15	1.01<br>.31	-1.29<br>.20	1.16<br>.25	0.39<br>.70	-0.40<br>.69
Sex × Valproate × threat omnibus	4.28*<br>.02	5.51*<br>.008	7.84**<br>.001	6.56*<br>.003	1.11<br>.32	5.01<br>.011	0.71<br>.48
Sex × Valproate × threat linear	2.55*<br>.01	2.65<br>.009	-3.42**<br>< .001	3.14*<br>.002	-0.53<br>.60	-2.35<br>.02	-1.06<br>.29
Sex × Pregabalin × threat omnibus	0.42<br>.63	0.60<br>.52	1.04<br>.34	0.70<br>.47	0.77<br>.45	0.64<br>.50	0.37<br>.68
Sex × Pregabalin × threat linear	-0.45<br>.65	0.11<br>.91	-0.55<br>.58	0.45<br>.65	0.85<br>.39	0.46<br>.65	0.25<br>.80
Sex × Valproate × task	1.05<br>.30	0.98<br>.33	0.64<br>.52	1.29<br>.20	-0.79<br>.43	0.15<br>.88	-1.83<br>.07
Sex × Pregabalin × task	0.23<br>.82	-0.12<br>.90	-0.79<br>.43	0.29<br>.78	0.34<br>.73	1.09<br>.28	-0.51<br>.61
Sex × Valproate × time omnibus	0.32<br>.68	1.37<br>.25	0.87<br>.46	1.89<br>.13	1.41<br>.24	0.89<br>.44	0.22<br>.80
Sex × Valproate × time linear	-0.51<br>.61	-1.73<br>.08	1.80<br>.07	-1.44<br>.15	2.21<br>.03	0.64<br>.52	-1.17<br>.24
Sex × Pregabalin × time omnibus	0.79<br>.43	1.12<br>.33	0.50<br>.69	0.70<br>.55	0.88<br>.45	0.43<br>.71	0.18<br>.83
Sex × Pregabalin × time linear	-3.07**<br>.003	-3.45**<br>< 0.001	1.73<br>.08	-2.47<br>.01	2.41<br>.02	2.04<br>.043	0.16<br>.87
Sex × Valproate × threat × time omnibus	0.78<br>.59	0.75<br>.62	1.62<br>.10	0.78<br>.65	0.96<br>.48	1.22<br>.25	0.85<br>.56
Sex × Valproate × threat × time linear	0.96<br>.33	0.51<br>.61	0.94<br>.35	-0.89<br>.38	-3.36**<br>< .001	1.01<br>.31	0.63<br>.53
Sex × Pregabalin × threat × time omnibus	0.66<br>.69	0.62<br>.73	1.00<br>.44	0.74<br>.68	0.71<br>.72	0.67<br>.82	1.22<br>.28
Sex × Pregabalin × threat × time linear	2.45*<br>.01	-0.26<br>.80	-0.12<br>.91	-0.12<br>.90	0.95<br>.34	-0.53<br>.60	-3.00*<br>.003

Results are shown from a 2 (Sex: Male, Female) × 3 (Threat: Low, Medium, High) × 2 (Task: Active/Passive) × 15 (Time bins of 1 s each) ANOVA. The table lists F-values for omnibus interactions and signed t-values for polynomial interactions and for interactions with drug and task. Only sex × drug interactions are shown. P-values were computed using Greenhouse-Geisser corrected degrees of freedom. Significant p-values after Bonferroni correction (primary outcome: α = .05; secondary outcome: α = .05/6 ≅ .008) are marked with *. Signs of t-values are coded as higher dependent values for females, for higher levels of threat, later time points, and passive vs. active. Shaded column: primary outcome.

Table S7: Comparison of Valproate/Pregabalin with 1 mg lorazepam.

	**Presence in safe place**	**Distance from threat**	**Distance from walls**	**Presence in safe quadrant**	**Presence in threat quadrant**	**Tokens per second**	**Speed when on grid**
	t	t	t	t	t	t	t
	P	*p*	*p*	*p*	*p*	*p*	*p*
All drugs vs placebo × time linear	-3.79*	-4.33*	0.29	-3.06*	3.68*	-1.98	-3.00*
	< .001	< .001	0.77	0.002	< .001	0.05	0.003
Current vs. previous study × time linear	2.45*	1.17	-2.87*	1.58	1.80	-3.33*	-3.60*
	0.02	0.24	0.004	0.11	0.07	< .001	< .001
Drug × study × time linear	-2.28*	-0.72	4.37*	-1.87	-2.13	5.53*	2.91*
	0.02	0.47	< .001	0.06	0.03	< .001	0.004

Results are shown from a 2 (Study: Valproate/Pregabalin vs. Lorazepam) × 2 (Group: Drug vs. Placebo) ×3 (Threat: Low, Medium, High) × 2 (Task: Active/Passive) × 15 (Time bins of 1 s each) ANOVA. The table lists signed t-values for the a priori contrasts (linear interactions with time). P-values were computed using Greenhouse-Geisser corrected degrees of freedom. Significant p-values after Bonferroni correction (primary outcome: $\alpha = .05$; secondary outcome: $\alpha = .05/6 \cong 0.008$) are marked with *. Signs of t-values are coded as higher dependent values for later time points, for the current study, and for drug. Shaded column: primary outcome.
