# Supplementary figures and images for: Effect of valproate and pregabalin on human anxiety-like behaviour in a randomised controlled trial
Source: Transl Psychiatry. 2018 Aug 16;8:157. doi: 10.1038/s41398-018-0206-7 (PMC6095858; doi:10.1038/s41398-018-0206-7)

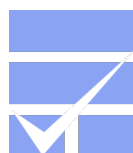

# CONSORT

TRANSPARENT REPORTING of TRIALS

## CONSORT 2010 Flow Diagram

### AAAX2 trial

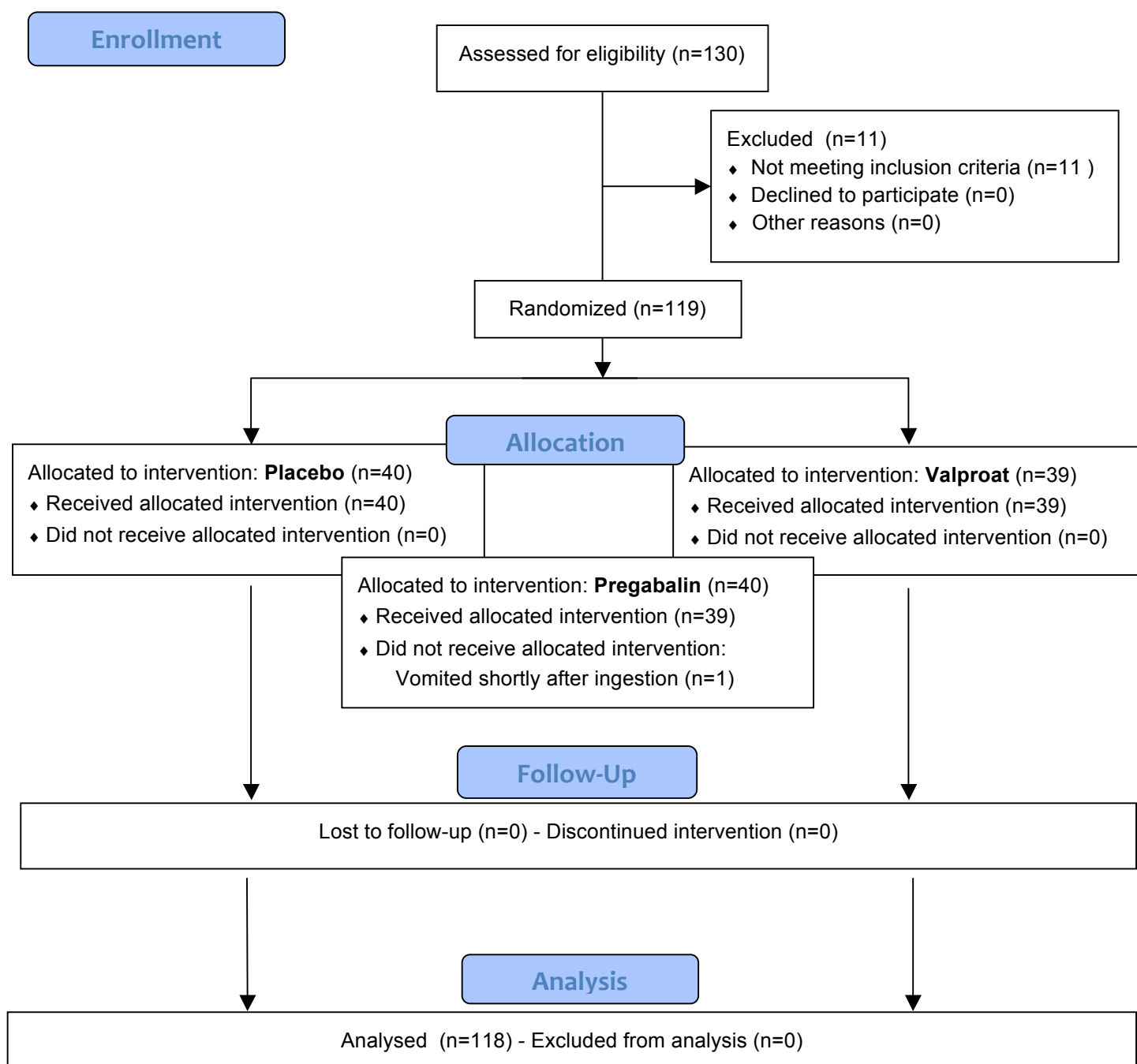

Supplement: Supplementary file 2 — Consort Flow Chart [file 41398_2018_206_MOESM2_ESM.pdf]
